# The Role of Biofilms in Contact Lens Associated Fungal Keratitis

**DOI:** 10.3390/antibiotics12101533

**Published:** 2023-10-12

**Authors:** Jipan Yi, Yao Sun, Chenghong Zeng, Xenia Kostoulias, Yue Qu

**Affiliations:** 1 Department of Optometry, Zhejiang Industry & Trade Vocational College, Wenzhou 325000, China; yijipan@zjitc.edu.cn (J.Y.); chenghongzeng@zjitc.edu.cn (C.Z.); 2 Monash Biomedicine Discovery Institute, Department of Microbiology, Monash University, Clayton, VIC 3800, Australia; yao.sun@monash.edu (Y.S.); xenia.kostoulias@monash.edu (X.K.); 3 Department of Infectious Diseases, The Alfred Hospital and Monash University, Clayton, VIC 3000, Australia

**Keywords:** CLAFK, fungal biofilms, pathogenesis, persistence, contact lens care system and solution, *Fusarium* species, *Aspergillus* species, *Candida* species

## Abstract

Biofilm formation is an important microbial strategy for fungal pathogens, such as *Fusarium*, *Aspergillus*, and *Candida*, to establish keratitis in patients wearing soft contact lenses. Despite the well-documented 2006 outbreak of *Fusarium* keratitis that eventually led to the withdrawal of the Bausch & Lomb multipurpose lens care solution ReNu with MoistureLoc (“MoistureLoc”) from the global market, contact lens care systems and solutions currently available on the market do not specifically target fungal biofilms. This is partially due to the lack of recognition and understanding of important roles that fungal biofilms play in contact lens associated fungal keratitis (CLAFK). This review aims to reemphasize the link between fungal biofilms and CLAFK, and deepen our comprehension of its importance in pathogenesis and persistence of this medical device-related infection.

## 1. Introduction

Fungal keratitis refers to microbial infections of the cornea caused by mycotic pathogens [1]. These infections mostly present fulminant clinical features and often lead to irreversible and devastating sequelae if correct diagnosis and timely treatment are delayed [2,3,4,5]. Due to its aggressive clinical traits, fungal keratitis is often associated with high patient morbidity, increased Medicare costs, excessive hospital-stays and often requires keratoplasty [1,6,7]. The most frequently encountered pathogens causing fungal keratitis include *Fusarium, Candida* and *Aspergillus* species [1,6,8,9,10]. In Asia and South America, *Fusarium* spp. are the predominant pathogen, responsible for ~50% of fungal keratitis cases, with most incidences associated with agricultural operations [8,11,12]. In the USA and Australia, *Candida albicans* has been reported as the most common pathogen causing fungal keratitis, particularly in patients with a history of chronic ocular surface and atopic diseases, topical steroid use, ocular trauma or contact lens (CL) wear [13,14].

Globally, CL wear has been identified as a major risk factor, substantially contributing to the incidence of fungal keratitis [9,15,16]. CL wear may induce hypoxia and mechanic microtrauma of the corneal epithelium; furthermore, CLs can serve as a substratum for fungal biofilm growth and facilitate fungal transmission from the environment to compromised corneal surfaces if handled and cared for improperly [17,18]. Biofilm is an important microbial growth mode that is associated with the occurrence and persistence of many medical device-related infections [19,20]. The important role of this special microbial growth mode in CL-related bacterial keratitis was proposed as early as the 1980s [21], while the link between biofilms and CL-associated fungal keratitis (CLAFK) was not recognized for the major pathogens *C. albicans* until 1991 [22] and *Fusarium* spp. until 2007 [23]. Despite the well-documented outbreak of *Fusarium* keratitis in 2006 [24], only a handful of studies have been conducted to evaluate the importance of fungal biofilms in CLAFK [23,25,26,27,28]. This mini-review focuses on our current comprehension of the importance of biofilm formation in the pathogenesis and persistence of CLAFK.

## 2. Clinical and Experimental Evidence Supports the Involvement of Fungal Biofilms in CLAFK

The involvement of fungal biofilms in CLAFK is supported by three tiers of clinical or experimental evidence. Firstly, leading pathogens of fungal keratitis are typical biofilm producers. *Fusarium* spp., *Aspergillus* spp., and *Candida albicans* isolated from patients with clinically diagnosed CLAFK have been reported to form in vitro biofilms either in tissue culture treated multi-well microplates, lens cases, various CLs and human corneal epithelial cells [25,27,28,29,30,31]. Secondly, evidence of fungal biofilms on patients’ CLs or in lens cases and on the cornea of different experimental animals have been observed. Numerous studies employing high-resolution scanning electron microscopy (SEM), fluorescent in situ hybridization (FISH) or confocal laser scanning microscopy (CLSM), in combination with fungus-specific probes, have detected fungal biofilms of diverse morphologies on the surface of CLs, lens cases or the cornea, including sporadic cells, monolayers, microcolonies and macro-colony biofilms [23,32,33]. Additionally, the presence of biofilm-like fungal populations in the cornea is often accompanied by corneal histopathological changes [33,34,35]. Lastly, the recurrent pattern of fungal keratitis after corneal transplantation and/or antifungal treatment coincides with the model of relapsing infections proposed by Lewis et al. (2010), in which biofilm formation is the major culprit [36,37,38,39].

## 3. Understanding the Structural Characteristics of Fungal Biofilms Related to CLAFK

Biofilm is a unique microbial growth mode with free-living or planktonic growth as its counterpart [40]. Biofilm formation is a survival strategy of microorganisms that is characterized by lowered metabolism of the general population, altered gene expression of embedded cells, and high tolerance to antimicrobial agents and host immune responses [41]. Most in vitro fungal biofilms, cultured in microbiology research laboratories using different substrata and incubation conditions [42,43,44,45], have complex structures comprising of cell aggregates encased within a self-produced matrix of extracellular polymeric substances (EPS). Such multicellular structures embedded within intercellular EPS were also observed when dimorphic fungi such as *C. albicans* or filamentous fungi such as *Fusarium* spp. were cultured on soft CLs in broth media for 48 h [25,46], *C. albicans* on human cadaveric cornea or *A. fumigatus* on primary cultures of human Limbo-Corneal fibroblasts for 24 h [47,48].

The presence of EPS matrix is the typical morphological characteristic of fungal biofilms. Fungal biofilm EPS matrix is often composed of extracellular polysaccharides and monosaccharides, major antigen and hydrophobins, proteins, melanin, lipids, phosphorus, uronic acid and extracellular DNA (eDNA) [49,50,51]. The composition of fungal biofilm EPS matrix differs among different species or strains at different growth stages [49,50,51,52]. Polysaccharides and proteins are generally considered as the principal components of fungal biofilm matrix, accounting for over 80% in relative abundance for abiotic biofilms formed by *C. albicans* and *A. fumigatus* [52]. Extracellular DNA is another important component of fungal biofilm matrix, which is at least partially acquired from autolysis of fungal cells within biofilms [53,54]. Polysaccharides and eDNA in the matrix are not only vital for fungal biofilm architectural integrity, but play an important role in biofilm resistance to antifungals and human immune responses [52,53,54].

## 4. In Vitro and In Vivo Modeling of Fungal Biofilm Formation to Reflect the Important Role of Pathogen-Biomaterial or Pathogen-Ocular Surface Interactions in CLAFK

Two types of fungal biofilms have been implicated in CLAFK, including abiotic biofilms that require a biomaterial-based substratum, such as the surface of CLs or lens cases, and tissue-based biotic biofilms that grow on or underneath the corneal epithelium [55,56]. Abiotic and biotic biofilms formed by the same fungal species may differ in their morphology and antifungal resistance [44]. Most silicone hydrogel soft CLs, pHEMA soft CLs, and Rigid Gas Permeable (RGP) lenses on the market inevitably support biofilm formation by leading fungal pathogens such as *Fusarium* spp. and *C. albicans* on their surfaces [25,57,58]. Several physiochemical characteristics of biomaterials may play determinant roles in the formation of abiotic biofilm in CLAFK, including surface chemistry, hydrophobicity and roughness of CLs and lens cases [59]. Numerous studies of other medical devices or biomaterials have found that surface chemistry played a more important role than roughness and hydrophobicity in the pathogen-biomaterial interactions and biofilm formation [45,60,61]. In vitro fungal biofilms have been established in multi-well polystyrene microplates, lens cases, and on CLs, in order to investigate fungus-biomaterial interactions encountered in CLAFK [62]. Using CLs stored in 12-well polystyrene microplates, it has been reported that CL polymers lotrafilcon A and polymacon supported greater biofilm formation of *F. solani* than other polymers including etafilcon A, galyfilcon A, balafilcon A, and alphafilcon A [25]. Simulating biotic fungal biofilms and the relevant fungus-cornea interactions appear to be more challenging. Isolated rabbit corneas, human cadaveric corneas, human corneal epithelial cell lines and primary limbo-corneal fibroblast monolayers have all been used as ex vivo or in vitro models for the formation of biotic fungal biofilms on corneal epithelial cells [30,48,63]. A widely-accepted CLAFK animal model assesses in vivo pathogen-ocular surface interactions by placing soft CLs with adhered *Fusarium* or *Aspergillus* cells on pre-injured mouse cornea and observing the progress of induced infections [33,64,65].

## 5. Transition from Abiotic Biofilms to Biotic Biofilms Plays an Important Role in the Pathogenesis of CLAFK

**Figure 1:**
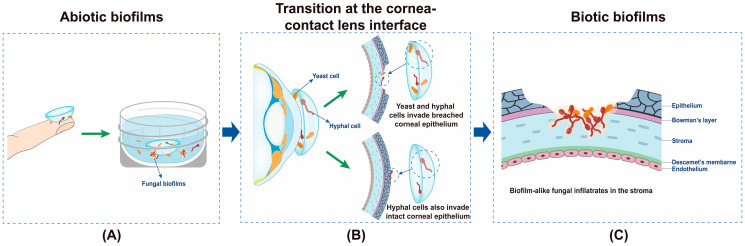
Transmission of fungal pathogens from lens care system to the human cornea. (**A**) Initial contamination of CLs and lens cases, (**B**) Fungal pathogens reach the proximity of human cornea and invade breached or even intact corneal epithelium, (**C**) Fungal cells reach stromal layer of the cornea and form biotic biofilms.

The pathogenesis of CLAFK has not been fully elucidated. A transition from abiotic biofilms in the CL care system to biotic biofilms on the cornea appears to be essential for the occurrence of CLAFK (Figure 1). To establish a CLAFK, fungal cells must possess certain virulence factors that are crucial for their survival within the CL care system and on the host corneal epithelium. This includes the ability to form self-protective biofilms and transition from abiotic to biotic surfaces. It has been proposed that fungal cells causing CLAFK originate from those residing in CL solutions or lens cases [23,66], using CLs as an intermediate mediator to reach the human cornea [67]. Proposedly, fungal cells from external environments contaminate CLs and lens cases, forming monolayer/microcolony biofilms on their surfaces that survive overnight CL disinfection [18,68] (Figure 1A). Small cell clusters from the biofilms can be brought to the proximity of the cornea and survive the dynamic and naturally antimicrobial ocular environment (Figure 1B). The coincidence of corneal erosions during lens wear may allow fungal cells to bypass corneal epithelial barriers such as the tight junctions (Figure 1B) [69]. Filamentous fungi also have the capability to directly invade corneal epithelial cells by induced endocytosis or hyphae-associated active penetration [70] (Figure 1B). Ultimately, fungal cells reach the corneal stroma and reside there as biotic biofilms and induce potent local inflammatory responses (Figure 1C).

## 6. Formation of Fungal Biofilms Contributes to the Persistence of CLAFK

It is well established that fungal keratitis cannot be completely prevented by strictly following recommended CL care and storage guidelines, and established infections often fail to respond to conservative antifungal treatment requiring surgical interventions such as therapeutic penetrating keratoplasty [71,72]. Based on published experimental data, it is reasonable to speculate that formation of abiotic and biotic biofilms contributes to fungal tolerance to CL cleaning and care, first-line antifungal drugs and ocular immune responses to fungal invasion [22,23,25,27,28,46,73].

An association between multi-drug resistance and biofilm formation has been well established for bacterial and fungal pathogens [74,75,76]. Fungal biofilms formed on CLs, lens cases, and ocular surfaces are not exceptions [22,23,25,27,28,46,73]. Although planktonically grown *Fusarium* and *C. albicans* cells remained sensitive to multipurpose CL solutions such as MoistureLoc and MultiPlus under standard testing conditions [25]; such solutions are only partially effective against fungal biofilms formed on CLs or lens cases [25]. For instance, Dosler et al. observed only a ~1.5 log colony-forming unit’s (CFU) reduction of the total 5.5 log CFU of biofilm cells grown on used CLs following overnight exposure to multipurpose CL solutions Renu or Opti Free [46]. Conversely, Retuerto and colleagues reported that CL solutions containing active disinfectants polyquaternium-1 and myristamidopropyl dimethylamine, or 3% hydrogen peroxide were effective against *Fusarium* biofilms formed on CLs or in CL cases [57]. In their study, the presence of viable filamentous cells might not have been accurately determined by the XTT method used in their study [77], supported by the very low OD492 readings for the untreated control biofilms [57].

The antifungal susceptibility of biotic fungal biofilms formed on the cornea has been rarely studied. Ex vivo rabbit and human corneas [63] and in vitro human tissue-engineered cornea [78] may serve as valuable research tools for this purpose. The mechanisms underlying the irresponsiveness of CL-related fungal biofilms to antifungal prevention or treatment are yet to be comprehensively studied. It is reasonable to infer that they would not differ significantly from those reported for other fungal biofilm-related infections, including the hindered penetration of antifungal agents to EPS matrix-embedded fungal cells, slow metabolism of densely packed fungal cells, and the presence of persister cells [79].

Scarcity of the antifungal arsenal has left very limited options for conservative treatment of CLAFK. Resistance to fluconazole in ocular isolates of *C. albicans* is common [80], possibly involving genes encoding or regulating the ergosterol biosynthesis pathway, drug transporters, changes in ploidy, and loss of heterozygosity [81]. Other common ocular fungal pathogens also share a high resistance rate to various azoles including itraconazole and voriconazole [82]. Amphotericin B is a very active antifungal drugs for ocular fungal infections [82]. Resistance to amphotericin B, however, is not an uncommon event for *Fusarium*, *Aspergillus*, and *Candida* strains isolated from patients with fungal keratitis [83]. Caspofungin has been recommended for treatment of ocular infections caused by azole or Amphotericin B resistant strains of *C. albicans* or other fungal species, though the emergence of caspofungin-resistant or intermediate-resistant fungal strains have also been reported [82]. This highlights the urgency of developing novel antifungals that bypass mechanisms underpinning the “conventional” and biofilm-associated resistance of fungal cells for the treatment of CLAFK.

Biofilm-embedded fungal cells may also be tolerant to ocular immune responses and host defenses. Fungal immune evasion in the ocular environment and the host defense mechanism of the cornea have been comprehensively reviewed by Fleiszig and Evans (2010) [84] and more recently by Mills et al. (2021) [7] Immune invasion of biotic fungal biofilms grown on/in the cornea and its underlying mechanisms are still inadequately understood. Research is largely needed to clarify the interaction of abiotic and biotic fungal biofilms with the host immune responses on the ocular surface.

## 7. Implications for Effective Biofilm-Specific Preventative Strategies

Suboptimal treatment outcomes of CLAFK highlights the importance of preventing its occurrence. Hygienic practice and strict compliance with the CL care guide have been recommended to minimize fungal contamination of CLs and lens cases. These include regularly replacing used CLs and lens cases that are more prone to fungal attachment to prevent planktonic cells forming more resistant adherent monolayers if contamination does occur [17]. Rubbing, cleaning and rinsing the lenses before overnight storage and wiping lens cases may also be important for prevention of CLAFK as they mechanically remove monolayer or microcolony biofilms grown on the contaminated CLs and cases. Unfortunately, hygienic practice of CL wear and care does not guarantee sufficient risk mitigation for CLAFK. It is also worth mentioning that filamentous fungi such as *F. solani* and *Ulocladium* species can penetrate the matrix of conventional and silicone hydrogel CLs [23,62]; whether filamentous fungal cells that have penetrated CL matrix can be removed by rubbing and rinsing remains unknown and warrants investigation.

Multipurpose CL solutions are widely used as a preventative strategy against CL-related microbial infections. Many multipurpose CL solutions attribute their antimicrobial efficacy to biocides or preservatives that are minimally effective against fungal cells, such as polyquaternium-1 (PQ-1) and biguanides (PHMB) [85]. Mendonca et al. recently assessed the anti-infectiveness of 14 multipurpose CL solutions against *Candida* biofilms pre-established in lens cases. They found that none satisfactorily reached the expected reduction cutoff, indicating the infectiveness of most CL solutions on the market against fungal biofilms [86]. Using biofilm-active antiseptics or preservatives as disinfectant components in the CL solutions may interrupt the transmission of fungal pathogens from the CL care system to the cornea. Dimethylamine, Povidone-iodine, and H_2_O_2_, and a combination of polyquaternium-1 and myristamidopropyl, have all demonstrated promising potential in preventing biofilm formation by ocular fungal isolates [10,22,57,68,85,87]. Optimizing the concentrations of these agents in the CL solutions for better anti-fungal-biofilm activities, either as a single antiseptic or combinational preservatives, will possibly see a reduced incidence rate of CLAFK. Novel anti-biofilm agents have also been introduced into the CL care systems to prevent CLAFK, such as enzymes or chemicals that can break down the EPS matrix of fungal biofilms, or novel inhibitors of quorum sensing systems that regulate fungal biofilm formation [47,88,89]. Although these novel agents demonstrated excellent anti-fungal-biofilm activities in vitro, they are still in early experimental stages and clinical success is yet to be reported [47].

## 8. Conclusions

Fungal biofilms are an important clinical entity, and are increasingly recognized as interkingdom structures alongside a myriad of bacterial species causing devastating eye infections when medical devices, such as CLs, are inserted. These difficult-to-prevent infections often respond poorly to antifungal drugs routinely used in clinical settings. The wider clinical and scientific community are now recognizing the importance of fungal biofilm formation in CLAFK. There is a significant lack of studies investigating the genetic characteristics of causative fungi of CLAFK, in particular their contribution to the formation of abiotic and biotic fungal biofilms. More fundamental and in vitro research, pre-clinical studies, and clinical trials are urgently needed to obtain a comprehensive understanding of the pathogenesis and persistence of CLAFK, and to develop more effective preventative and treatment strategies for this devastating ocular infection.

## Data Availability

Not applicable.
